# (*E*)-1-(3-Hy­droxy­phen­yl)-3-[4-(tetra­dec­yl­oxy)phen­yl]prop-2-en-1-one

**DOI:** 10.1107/S1600536812038020

**Published:** 2012-09-08

**Authors:** Siti Muhaini Haris Fadzillah, Zainab Ngaini, Hasnain Hussain, Ibrahim Abdul Razak, Safra Izuani Jama Asik

**Affiliations:** a Department of Chemistry, Faculty of Resource Science and Technology, Universiti Malaysia Sarawak, 94300 Kota Samarahan, Sarawak, Malaysia; b Department of Molecular Biology, Faculty of Resource Science and Technology, Universiti Malaysia Sarawak, 94300 Kota Samarahan, Sarawak, Malaysia; c School of Physics, Universiti Sains Malaysia, 11800 USM, Penang, Malaysia

## Abstract

In the title compound, C_29_H_40_O_3_, the enone moiety adopts an *s-cis* conformation. The dihedral angle between the benzene rings is 4.33 (5)° The least-squares mean line through the tetra­decyl side chain forms a dihedral angle of 83.99 (7)° with the normal to the attached benzene ring. In the crystal, O—H⋯O and C—H⋯O hydrogen bonds involving the keto and the hy­droxy O atoms form ribbons along [-41-1]. The crystal structure also features C—H⋯π inter­actions.

## Related literature

For the biological properties of chalcone derivatives, see: Bhat *et al.* (2005 ▶); Xue *et al.* (2004 ▶); Won *et al.* (2005 ▶); Zhao *et al.* (2005 ▶); Satyanarayana *et al.* (2004 ▶). For related structures, see: Razak *et al.* (2009 ▶); Ngaini *et al.* (2010 ▶, 2011 ▶). For the stability of the temperature controller used in the data collection, see: Cosier & Glazer (1986 ▶). For bond-length data, see: Allen *et al.* (1987 ▶).
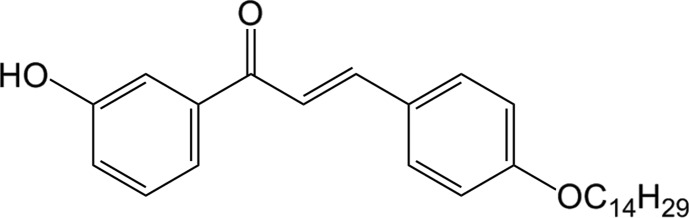


## Experimental

### 

#### Crystal data

C_29_H_40_O_3_
*M* *_r_* = 436.61
Triclinic, 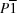

*a* = 6.5138 (16) Å
*b* = 10.155 (2) Å
*c* = 19.264 (5) Å
α = 75.361 (6)°
β = 85.872 (7)°
γ = 83.013 (6)°
*V* = 1222.6 (5) Å^3^
*Z* = 2
Mo *K*α radiation
μ = 0.08 mm^−1^
*T* = 100 K
0.29 × 0.12 × 0.08 mm

#### Data collection

Bruker APEX DUO CCD area-detector diffractometer
Absorption correction: multi-scan (*SADABS*; Bruker, 2009 ▶) *T*_min_ = 0.979, *T*_max_ = 0.994
26295 measured reflections
7155 independent reflections
5052 reflections with *I* > 2σ(*I*)
*R*_int_ = 0.037

#### Refinement

*R*[*F* ^2^ > 2σ(*F* ^2^)] = 0.046
*wR*(*F* ^2^) = 0.147
*S* = 0.95
7155 reflections
293 parameters
H atoms treated by a mixture of independent and constrained refinement
Δρ_max_ = 0.40 e Å^−3^
Δρ_min_ = −0.23 e Å^−3^

### 

Data collection: *APEX2* (Bruker, 2009 ▶); cell refinement: *SAINT* (Bruker, 2009 ▶); data reduction: *SAINT* (Bruker, 2009 ▶); program(s) used to solve structure: *SHELXTL* (Sheldrick, 2008 ▶); program(s) used to refine structure: *SHELXTL* (Sheldrick, 2008 ▶); molecular graphics: *SHELXTL* (Sheldrick, 2008 ▶); software used to prepare material for publication: *SHELXTL* (Sheldrick, 2008 ▶) and *PLATON* (Spek, 2009 ▶).

## Supplementary Material

Crystal structure: contains datablock(s) global, I. DOI: 10.1107/S1600536812038020/rz5001sup1.cif

Structure factors: contains datablock(s) I. DOI: 10.1107/S1600536812038020/rz5001Isup2.hkl

Supplementary material file. DOI: 10.1107/S1600536812038020/rz5001Isup3.cml

Additional supplementary materials:  crystallographic information; 3D view; checkCIF report

## Figures and Tables

**Table 1:** Hydrogen-bond geometry (Å, °) *Cg*1 and *Cg*2 are the centroids of the C10–C15 and C1–C6 rings, respectively.

*D*—H⋯*A*	*D*—H	H⋯*A*	*D*⋯*A*	*D*—H⋯*A*
O1—H1*O*1⋯O2^i^	0.93 (2)	1.80 (2)	2.7269 (14)	175.6 (18)
C29—H29*A*⋯O1^ii^	0.96	2.44	3.3589 (18)	160
C17—H17*B*⋯*Cg*1^iii^	0.97	2.73	3.6159 (16)	152
C28—H28*A*⋯*Cg*2^iv^	0.97	2.93	3.8481 (16)	159

Symmetry codes: (i) 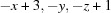; (ii) 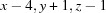; (iii) 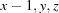; (iv) 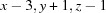.

**Fig. 1.**
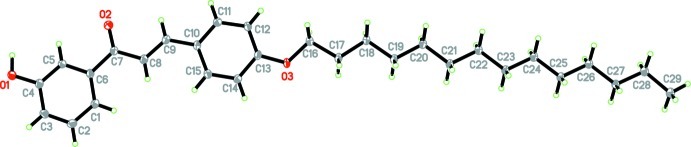
The structure of the title compound, showing 50% probability displacement ellipsoids.

**Fig. 2.**
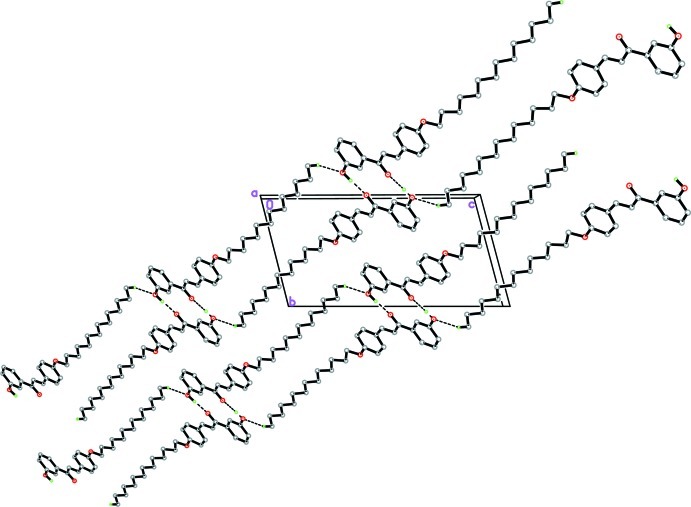
Packing diagram of the title compound viewed down the a axis, showing the alternately interconnected extended chains parallel to the [4 -1 1] direction. Hydrogen atoms not involved in hydrogen bonds (dashed lines) are omitted.

## supplementary crystallographic information

### Comment

Chalcones are highly reactive subtances of varied nature. They have been reported to possess many useful properties including anti-malarial (Xue *et al.*, 2004), anti-cancer (Bhat *et al.*, 2005), anti-inflammatory (Won *et al.*, 2005), anti-platelet (Zhao *et al.*, 2005) and anti-hyperglynemic (Satyanarayana *et al.*, 2004) activities. Herein, we report the crystal structure of the title compound (Fig. 1).

The enone moiety (O2/C7–C9) adopts an *s-cis* conformation with the O2–C7–C8–C9 torsion angle of 0.64 (17)°. The dihedral angles between the least-square plane through the enone moiety and the benzene rings (C1–C6 and C10–C15) are 6.26 (7) and 4.65 (7)°, respectively. The dihedral angle between these benzene rings is 4.33 (5)°. The bond lengths observed in the title compound are comparable with the values previously reported values in the literature (Allen *et al.*, 1987).

The short H8A···H15A (2.20 Å) and H8A···H1A (2.11 Å) contacts results in the widening of C8–C9–C10 (126.84 (11)°) and C1–C6–C7 (123.31 (10)°) angles, respectively. The geometric parameters are consistent to those observed in closely related structures (Razak *et al.*, 2009; Ngaini *et al.*, 2010; Ngaini *et al.*, 2011).

The conformation throughout the zigzag alkoxyl tail is *trans* and is roughly coplanar with the attached benzene (C10–C15) ring as the torsion angle C16–O3–C13–C14 is 176.57 (10)°. However, only the aliphatic part (C16–C29) of the alkoxyl tail is constantly within the zigzag plane. The torsion angle of the aliphatic part deviate from the ideal value by 0.02 (10)–3.75 (10)° while the O3–C16–C17–C18 torsion angle shows value of 173.64 (9)°.

In the crystal packing (Fig. 2), the molecules are arranged in head-to-tail manner along the [-4 1 -1] direction. This arrangement is linked into extended chains through C29—H29···O1 intermolecular interactions. These chains are alternately interconnected by O1—H1O1···O2 intermolecuar hydrogen bonds. Furthermore, the crystal packing is stabilized by weak C—H···π interactions (Table 1) with the distance of 3.6159 (16) and 3.8481 (16) Å.

### Experimental

A mixture of 3-hydroxyacetophenone (1.36 g, 10 mmol) and 4-tetradecyloxybenzaldehyde (3.19 ml, 10 mmol) in methanol (40 ml) was heated at reflux for 12 h. The reaction was cooled to room temperature and acidified with cold diluted HCl (2N). The resulting precipitate was filtered, washed and dried. After redissolving in hexane-ethanol (7:1 *v*/*v*) followed by few days of slow evaporation, crystals were collected.

### Refinement

The O-bound H atom was located in a difference Fourier map and refined freely with O–H = 0.927 (19) Å. The remaining H atoms were placed in calculated positions with C–H = 0.93–0.97 Å. The *U*_iso_ values were constrained to be 1.5*U*_eq_(C) for methyl H atoms and 1.2*U*_eq_(C) for other H atoms. The rotating model group was applied to the methyl group. Two outliers (0 0 1) and (1 0 1) were omitted.

### Crystal data

**Table:**

C_29_H_40_O_3_	*Z* = 2
*M**_r_* = 436.61	*F*(000) = 476
Triclinic, *P*1	*D*_x_ = 1.186 Mg m^−^^3^
Hall symbol: -P 1	Mo *K*α radiation, λ = 0.71073 Å
*a* = 6.5138 (16) Å	Cell parameters from 5533 reflections
*b* = 10.155 (2) Å	θ = 2.6–30.1°
*c* = 19.264 (5) Å	µ = 0.08 mm^−^^1^
α = 75.361 (6)°	*T* = 100 K
β = 85.872 (7)°	Block, colourless
γ = 83.013 (6)°	0.29 × 0.12 × 0.08 mm
*V* = 1222.6 (5) Å^3^	

### Data collection

**Table:**

Bruker APEX DUO CCD area-detector diffractometer	7155 independent reflections
Radiation source: fine-focus sealed tube	5052 reflections with *I* > 2σ(*I*)
Graphite monochromator	*R*_int_ = 0.037
φ and ω scans	θ_max_ = 30.2°, θ_min_ = 2.1°
Absorption correction: multi-scan (*SADABS*; Bruker, 2009)	*h* = −9→9
*T*_min_ = 0.979, *T*_max_ = 0.994	*k* = −14→14
26295 measured reflections	*l* = −27→27

### Refinement

**Table:**

Refinement on *F*^2^	Primary atom site location: structure-invariant direct methods
Least-squares matrix: full	Secondary atom site location: difference Fourier map
*R*[*F*^2^ > 2σ(*F*^2^)] = 0.046	Hydrogen site location: inferred from neighbouring sites
*wR*(*F*^2^) = 0.147	H atoms treated by a mixture of independent and constrained refinement
*S* = 0.95	*w* = 1/[σ^2^(*F*_o_^2^) + (0.0913*P*)^2^ + 0.1348*P*] where *P* = (*F*_o_^2^ + 2*F*_c_^2^)/3
7155 reflections	(Δ/σ)_max_ < 0.001
293 parameters	Δρ_max_ = 0.40 e Å^−^^3^
0 restraints	Δρ_min_ = −0.23 e Å^−^^3^

### Special details

**Table:**

Experimental. The crystal was placed in the cold stream of an Oxford Cryosystems Cobra open-flow nitrogen cryostat (Cosier & Glazer, 1986) operating at 100.0 (1) K.
Geometry. All esds (except the esd in the dihedral angle between two l.s. planes) are estimated using the full covariance matrix. The cell esds are taken into account individually in the estimation of esds in distances, angles and torsion angles; correlations between esds in cell parameters are only used when they are defined by crystal symmetry. An approximate (isotropic) treatment of cell esds is used for estimating esds involving l.s. planes.
Refinement. Refinement of F^2^ against ALL reflections. The weighted R-factor wR and goodness of fit S are based on F^2^, conventional R-factors R are based on F, with F set to zero for negative F^2^. The threshold expression of F^2^ > 2sigma(F^2^) is used only for calculating R-factors(gt) etc. and is not relevant to the choice of reflections for refinement. R-factors based on F^2^ are statistically about twice as large as those based on F, and R- factors based on ALL data will be even larger.

### Fractional atomic coordinates and isotropic or equivalent isotropic displacement parameters (Å^2^)

**Table:**

	*x*	*y*	*z*	*U*_iso_*/*U*_eq_	
O1	1.67428 (14)	0.11123 (9)	0.62770 (5)	0.0245 (2)	
O2	1.18019 (13)	0.07397 (8)	0.44966 (4)	0.01891 (19)	
O3	0.03940 (13)	0.44359 (8)	0.27772 (4)	0.01874 (19)	
C1	1.10873 (19)	0.31949 (12)	0.55966 (6)	0.0187 (2)	
H1A	0.9815	0.3669	0.5452	0.022*	
C2	1.2188 (2)	0.35854 (12)	0.60903 (6)	0.0210 (3)	
H2A	1.1648	0.4326	0.6273	0.025*	
C3	1.4073 (2)	0.28868 (12)	0.63122 (6)	0.0193 (2)	
H3A	1.4798	0.3158	0.6642	0.023*	
C4	1.48910 (19)	0.17725 (11)	0.60411 (6)	0.0168 (2)	
C5	1.37933 (18)	0.13808 (11)	0.55490 (6)	0.0159 (2)	
H5A	1.4331	0.0635	0.5370	0.019*	
C6	1.19002 (18)	0.20879 (11)	0.53191 (5)	0.0147 (2)	
C7	1.08792 (18)	0.16453 (11)	0.47562 (5)	0.0149 (2)	
C8	0.88422 (19)	0.23100 (11)	0.45154 (6)	0.0175 (2)	
H8A	0.8178	0.2985	0.4726	0.021*	
C9	0.79155 (18)	0.19653 (11)	0.39981 (6)	0.0157 (2)	
H9A	0.8609	0.1263	0.3814	0.019*	
C10	0.59375 (18)	0.25740 (11)	0.36941 (5)	0.0148 (2)	
C11	0.51656 (19)	0.20878 (11)	0.31596 (6)	0.0165 (2)	
H11A	0.5911	0.1353	0.3017	0.020*	
C12	0.33214 (19)	0.26639 (11)	0.28337 (6)	0.0162 (2)	
H12A	0.2838	0.2318	0.2480	0.019*	
C13	0.22125 (18)	0.37651 (11)	0.30454 (5)	0.0151 (2)	
C14	0.29544 (19)	0.42667 (11)	0.35819 (6)	0.0167 (2)	
H14A	0.2211	0.5005	0.3722	0.020*	
C15	0.47706 (18)	0.36780 (11)	0.39023 (6)	0.0163 (2)	
H15A	0.5235	0.4015	0.4262	0.020*	
C16	−0.04781 (19)	0.40731 (11)	0.21970 (6)	0.0169 (2)	
H16A	−0.0784	0.3128	0.2336	0.020*	
H16B	0.0467	0.4194	0.1779	0.020*	
C17	−0.24440 (19)	0.50374 (11)	0.20419 (6)	0.0166 (2)	
H17A	−0.2112	0.5967	0.1981	0.020*	
H17B	−0.3398	0.4836	0.2456	0.020*	
C18	−0.35328 (18)	0.49674 (11)	0.13800 (6)	0.0166 (2)	
H18A	−0.3893	0.4046	0.1436	0.020*	
H18B	−0.2605	0.5182	0.0960	0.020*	
C19	−0.54867 (18)	0.59744 (11)	0.12739 (6)	0.0167 (2)	
H19A	−0.6427	0.5718	0.1687	0.020*	
H19B	−0.5117	0.6878	0.1259	0.020*	
C20	−0.66224 (19)	0.60539 (11)	0.05978 (6)	0.0171 (2)	
H20A	−0.5696	0.6318	0.0181	0.021*	
H20B	−0.7009	0.5155	0.0610	0.021*	
C21	−0.85585 (18)	0.70750 (11)	0.05220 (6)	0.0169 (2)	
H21A	−0.8168	0.7965	0.0525	0.020*	
H21B	−0.9489	0.6796	0.0936	0.020*	
C22	−0.97131 (19)	0.72124 (11)	−0.01577 (6)	0.0169 (2)	
H22A	−0.8797	0.7513	−0.0573	0.020*	
H22B	−1.0086	0.6321	−0.0167	0.020*	
C23	−1.16618 (18)	0.82165 (11)	−0.02125 (6)	0.0174 (2)	
H23A	−1.2582	0.7907	0.0200	0.021*	
H23B	−1.1288	0.9103	−0.0195	0.021*	
C24	−1.28162 (19)	0.83808 (11)	−0.08925 (6)	0.0175 (2)	
H24A	−1.3187	0.7494	−0.0912	0.021*	
H24B	−1.1901	0.8696	−0.1306	0.021*	
C25	−1.47682 (19)	0.93813 (11)	−0.09405 (6)	0.0178 (2)	
H25A	−1.4395	1.0267	−0.0921	0.021*	
H25B	−1.5681	0.9066	−0.0526	0.021*	
C26	−1.59373 (19)	0.95536 (11)	−0.16197 (6)	0.0173 (2)	
H26A	−1.5028	0.9869	−0.2035	0.021*	
H26B	−1.6319	0.8670	−0.1639	0.021*	
C27	−1.78807 (19)	1.05606 (12)	−0.16597 (6)	0.0189 (2)	
H27A	−1.7484	1.1453	−0.1665	0.023*	
H27B	−1.8745	1.0272	−0.1229	0.023*	
C28	−1.9153 (2)	1.06977 (12)	−0.23116 (6)	0.0193 (2)	
H28A	−1.8286	1.0958	−0.2744	0.023*	
H28B	−1.9611	0.9817	−0.2298	0.023*	
C29	−2.1028 (2)	1.17540 (13)	−0.23412 (7)	0.0252 (3)	
H29A	−2.1781	1.1808	−0.2760	0.038*	
H29B	−2.0581	1.2631	−0.2364	0.038*	
H29C	−2.1907	1.1490	−0.1919	0.038*	
H1O1	1.718 (3)	0.046 (2)	0.6024 (10)	0.056 (5)*	

### Atomic displacement parameters (Å^2^)

**Table:**

	*U*^11^	*U*^22^	*U*^33^	*U*^12^	*U*^13^	*U*^23^
O1	0.0180 (5)	0.0297 (5)	0.0300 (4)	0.0089 (4)	−0.0123 (4)	−0.0174 (4)
O2	0.0174 (5)	0.0197 (4)	0.0206 (4)	0.0027 (3)	−0.0034 (3)	−0.0084 (3)
O3	0.0155 (4)	0.0225 (4)	0.0195 (4)	0.0050 (3)	−0.0090 (3)	−0.0086 (3)
C1	0.0145 (6)	0.0203 (5)	0.0213 (5)	0.0036 (4)	−0.0041 (4)	−0.0069 (4)
C2	0.0192 (6)	0.0203 (5)	0.0260 (5)	0.0029 (5)	−0.0033 (5)	−0.0120 (4)
C3	0.0175 (6)	0.0214 (5)	0.0217 (5)	−0.0006 (5)	−0.0051 (4)	−0.0100 (4)
C4	0.0136 (6)	0.0188 (5)	0.0180 (5)	0.0007 (4)	−0.0030 (4)	−0.0050 (4)
C5	0.0150 (6)	0.0164 (5)	0.0170 (5)	0.0010 (4)	−0.0030 (4)	−0.0059 (4)
C6	0.0137 (6)	0.0160 (5)	0.0144 (4)	−0.0008 (4)	−0.0018 (4)	−0.0035 (4)
C7	0.0145 (6)	0.0150 (5)	0.0145 (4)	−0.0007 (4)	−0.0021 (4)	−0.0026 (4)
C8	0.0149 (6)	0.0190 (5)	0.0185 (5)	0.0021 (4)	−0.0031 (4)	−0.0058 (4)
C9	0.0143 (6)	0.0150 (5)	0.0167 (5)	0.0005 (4)	−0.0022 (4)	−0.0026 (4)
C10	0.0126 (6)	0.0160 (5)	0.0148 (4)	−0.0005 (4)	−0.0020 (4)	−0.0019 (4)
C11	0.0157 (6)	0.0171 (5)	0.0171 (5)	0.0008 (4)	−0.0016 (4)	−0.0059 (4)
C12	0.0149 (6)	0.0186 (5)	0.0157 (4)	0.0001 (4)	−0.0036 (4)	−0.0056 (4)
C13	0.0122 (5)	0.0168 (5)	0.0154 (4)	0.0013 (4)	−0.0034 (4)	−0.0027 (4)
C14	0.0148 (6)	0.0177 (5)	0.0183 (5)	0.0018 (4)	−0.0025 (4)	−0.0070 (4)
C15	0.0151 (6)	0.0184 (5)	0.0159 (5)	−0.0004 (4)	−0.0037 (4)	−0.0052 (4)
C16	0.0159 (6)	0.0189 (5)	0.0165 (5)	0.0008 (4)	−0.0058 (4)	−0.0051 (4)
C17	0.0140 (6)	0.0179 (5)	0.0172 (5)	0.0016 (4)	−0.0042 (4)	−0.0037 (4)
C18	0.0140 (6)	0.0177 (5)	0.0179 (5)	0.0018 (4)	−0.0052 (4)	−0.0043 (4)
C19	0.0139 (6)	0.0182 (5)	0.0173 (5)	0.0019 (4)	−0.0037 (4)	−0.0042 (4)
C20	0.0150 (6)	0.0185 (5)	0.0178 (5)	0.0023 (4)	−0.0043 (4)	−0.0051 (4)
C21	0.0147 (6)	0.0189 (5)	0.0167 (5)	0.0018 (4)	−0.0041 (4)	−0.0046 (4)
C22	0.0154 (6)	0.0182 (5)	0.0171 (5)	0.0013 (4)	−0.0048 (4)	−0.0046 (4)
C23	0.0154 (6)	0.0198 (5)	0.0166 (5)	0.0025 (4)	−0.0049 (4)	−0.0047 (4)
C24	0.0166 (6)	0.0180 (5)	0.0175 (5)	0.0022 (4)	−0.0050 (4)	−0.0043 (4)
C25	0.0166 (6)	0.0184 (5)	0.0181 (5)	0.0030 (4)	−0.0056 (4)	−0.0050 (4)
C26	0.0170 (6)	0.0174 (5)	0.0175 (5)	0.0018 (4)	−0.0056 (4)	−0.0044 (4)
C27	0.0190 (6)	0.0181 (5)	0.0202 (5)	0.0030 (5)	−0.0077 (4)	−0.0064 (4)
C28	0.0189 (6)	0.0216 (5)	0.0177 (5)	0.0021 (5)	−0.0063 (4)	−0.0058 (4)
C29	0.0220 (7)	0.0282 (6)	0.0269 (6)	0.0062 (5)	−0.0112 (5)	−0.0107 (5)

### Geometric parameters (Å, º)

**Table:**

O1—C4	1.3561 (14)	C18—C19	1.5239 (15)
O1—H1O1	0.927 (19)	C18—H18A	0.9700
O2—C7	1.2296 (13)	C18—H18B	0.9700
O3—C13	1.3594 (13)	C19—C20	1.5237 (15)
O3—C16	1.4346 (13)	C19—H19A	0.9700
C1—C2	1.3906 (16)	C19—H19B	0.9700
C1—C6	1.3958 (14)	C20—C21	1.5228 (15)
C1—H1A	0.9300	C20—H20A	0.9700
C2—C3	1.3799 (17)	C20—H20B	0.9700
C2—H2A	0.9300	C21—C22	1.5244 (15)
C3—C4	1.3959 (15)	C21—H21A	0.9700
C3—H3A	0.9300	C21—H21B	0.9700
C4—C5	1.3876 (15)	C22—C23	1.5205 (16)
C5—C6	1.3910 (16)	C22—H22A	0.9700
C5—H5A	0.9300	C22—H22B	0.9700
C6—C7	1.4977 (15)	C23—C24	1.5199 (15)
C7—C8	1.4642 (16)	C23—H23A	0.9700
C8—C9	1.3394 (15)	C23—H23B	0.9700
C8—H8A	0.9300	C24—C25	1.5208 (16)
C9—C10	1.4550 (16)	C24—H24A	0.9700
C9—H9A	0.9300	C24—H24B	0.9700
C10—C11	1.3960 (14)	C25—C26	1.5224 (15)
C10—C15	1.4041 (15)	C25—H25A	0.9700
C11—C12	1.3898 (16)	C25—H25B	0.9700
C11—H11A	0.9300	C26—C27	1.5210 (16)
C12—C13	1.3892 (14)	C26—H26A	0.9700
C12—H12A	0.9300	C26—H26B	0.9700
C13—C14	1.3996 (14)	C27—C28	1.5220 (15)
C14—C15	1.3728 (15)	C27—H27A	0.9700
C14—H14A	0.9300	C27—H27B	0.9700
C15—H15A	0.9300	C28—C29	1.5186 (17)
C16—C17	1.5133 (15)	C28—H28A	0.9700
C16—H16A	0.9700	C28—H28B	0.9700
C16—H16B	0.9700	C29—H29A	0.9600
C17—C18	1.5238 (15)	C29—H29B	0.9600
C17—H17A	0.9700	C29—H29C	0.9600
C17—H17B	0.9700		
			
C4—O1—H1O1	109.7 (12)	C20—C19—H19A	108.6
C13—O3—C16	120.08 (8)	C18—C19—H19A	108.6
C2—C1—C6	119.72 (11)	C20—C19—H19B	108.6
C2—C1—H1A	120.1	C18—C19—H19B	108.6
C6—C1—H1A	120.1	H19A—C19—H19B	107.5
C3—C2—C1	120.77 (10)	C21—C20—C19	112.36 (9)
C3—C2—H2A	119.6	C21—C20—H20A	109.1
C1—C2—H2A	119.6	C19—C20—H20A	109.1
C2—C3—C4	119.90 (10)	C21—C20—H20B	109.1
C2—C3—H3A	120.1	C19—C20—H20B	109.1
C4—C3—H3A	120.1	H20A—C20—H20B	107.9
O1—C4—C5	122.73 (9)	C20—C21—C22	114.06 (9)
O1—C4—C3	117.87 (10)	C20—C21—H21A	108.7
C5—C4—C3	119.40 (10)	C22—C21—H21A	108.7
C4—C5—C6	120.95 (10)	C20—C21—H21B	108.7
C4—C5—H5A	119.5	C22—C21—H21B	108.7
C6—C5—H5A	119.5	H21A—C21—H21B	107.6
C5—C6—C1	119.26 (10)	C23—C22—C21	112.97 (9)
C5—C6—C7	117.38 (9)	C23—C22—H22A	109.0
C1—C6—C7	123.31 (10)	C21—C22—H22A	109.0
O2—C7—C8	121.41 (10)	C23—C22—H22B	109.0
O2—C7—C6	118.66 (10)	C21—C22—H22B	109.0
C8—C7—C6	119.91 (9)	H22A—C22—H22B	107.8
C9—C8—C7	121.35 (10)	C24—C23—C22	113.65 (9)
C9—C8—H8A	119.3	C24—C23—H23A	108.8
C7—C8—H8A	119.3	C22—C23—H23A	108.8
C8—C9—C10	126.83 (10)	C24—C23—H23B	108.8
C8—C9—H9A	116.6	C22—C23—H23B	108.8
C10—C9—H9A	116.6	H23A—C23—H23B	107.7
C11—C10—C15	117.57 (10)	C23—C24—C25	113.25 (9)
C11—C10—C9	119.95 (10)	C23—C24—H24A	108.9
C15—C10—C9	122.45 (10)	C25—C24—H24A	108.9
C12—C11—C10	122.27 (10)	C23—C24—H24B	108.9
C12—C11—H11A	118.9	C25—C24—H24B	108.9
C10—C11—H11A	118.9	H24A—C24—H24B	107.7
C13—C12—C11	118.83 (10)	C24—C25—C26	113.70 (9)
C13—C12—H12A	120.6	C24—C25—H25A	108.8
C11—C12—H12A	120.6	C26—C25—H25A	108.8
O3—C13—C12	125.69 (10)	C24—C25—H25B	108.8
O3—C13—C14	114.44 (9)	C26—C25—H25B	108.8
C12—C13—C14	119.86 (10)	H25A—C25—H25B	107.7
C15—C14—C13	120.53 (10)	C27—C26—C25	112.98 (9)
C15—C14—H14A	119.7	C27—C26—H26A	109.0
C13—C14—H14A	119.7	C25—C26—H26A	109.0
C14—C15—C10	120.92 (10)	C27—C26—H26B	109.0
C14—C15—H15A	119.5	C25—C26—H26B	109.0
C10—C15—H15A	119.5	H26A—C26—H26B	107.8
O3—C16—C17	104.86 (8)	C26—C27—C28	114.37 (9)
O3—C16—H16A	110.8	C26—C27—H27A	108.7
C17—C16—H16A	110.8	C28—C27—H27A	108.7
O3—C16—H16B	110.8	C26—C27—H27B	108.7
C17—C16—H16B	110.8	C28—C27—H27B	108.7
H16A—C16—H16B	108.9	H27A—C27—H27B	107.6
C16—C17—C18	114.84 (9)	C29—C28—C27	112.26 (9)
C16—C17—H17A	108.6	C29—C28—H28A	109.2
C18—C17—H17A	108.6	C27—C28—H28A	109.2
C16—C17—H17B	108.6	C29—C28—H28B	109.2
C18—C17—H17B	108.6	C27—C28—H28B	109.2
H17A—C17—H17B	107.5	H28A—C28—H28B	107.9
C17—C18—C19	110.54 (9)	C28—C29—H29A	109.5
C17—C18—H18A	109.5	C28—C29—H29B	109.5
C19—C18—H18A	109.5	H29A—C29—H29B	109.5
C17—C18—H18B	109.5	C28—C29—H29C	109.5
C19—C18—H18B	109.5	H29A—C29—H29C	109.5
H18A—C18—H18B	108.1	H29B—C29—H29C	109.5
C20—C19—C18	114.83 (9)		
			
C6—C1—C2—C3	−0.31 (19)	C16—O3—C13—C12	−3.73 (17)
C1—C2—C3—C4	−0.14 (19)	C16—O3—C13—C14	176.57 (10)
C2—C3—C4—O1	−179.67 (11)	C11—C12—C13—O3	179.88 (11)
C2—C3—C4—C5	0.08 (18)	C11—C12—C13—C14	−0.44 (17)
O1—C4—C5—C6	−179.82 (11)	O3—C13—C14—C15	179.62 (10)
C3—C4—C5—C6	0.43 (18)	C12—C13—C14—C15	−0.09 (18)
C4—C5—C6—C1	−0.88 (18)	C13—C14—C15—C10	0.82 (18)
C4—C5—C6—C7	176.62 (10)	C11—C10—C15—C14	−0.98 (17)
C2—C1—C6—C5	0.81 (18)	C9—C10—C15—C14	177.11 (11)
C2—C1—C6—C7	−176.53 (11)	C13—O3—C16—C17	−179.19 (9)
C5—C6—C7—O2	−3.99 (16)	O3—C16—C17—C18	173.04 (9)
C1—C6—C7—O2	173.39 (11)	C16—C17—C18—C19	179.95 (10)
C5—C6—C7—C8	177.31 (10)	C17—C18—C19—C20	176.22 (10)
C1—C6—C7—C8	−5.30 (17)	C18—C19—C20—C21	179.98 (10)
O2—C7—C8—C9	−0.64 (18)	C19—C20—C21—C22	178.56 (10)
C6—C7—C8—C9	178.02 (11)	C20—C21—C22—C23	178.85 (10)
C7—C8—C9—C10	−177.78 (11)	C21—C22—C23—C24	179.19 (10)
C8—C9—C10—C11	−179.31 (12)	C22—C23—C24—C25	179.71 (10)
C8—C9—C10—C15	2.64 (19)	C23—C24—C25—C26	−179.99 (10)
C15—C10—C11—C12	0.44 (17)	C24—C25—C26—C27	−179.83 (10)
C9—C10—C11—C12	−177.70 (11)	C25—C26—C27—C28	−176.74 (10)
C10—C11—C12—C13	0.25 (18)	C26—C27—C28—C29	−177.78 (11)

### Hydrogen-bond geometry (Å, º)

Cg1 and Cg2 are the centroids of the C10–C15 and C1–C6 rings, respectively.

**Table:**

*D*—H···*A*	*D*—H	H···*A*	*D*···*A*	*D*—H···*A*
O1—H1*O*1···O2^i^	0.93 (2)	1.80 (2)	2.7269 (14)	175.6 (18)
C29—H29*A*···O1^ii^	0.96	2.44	3.3589 (18)	160
C17—H17*B*···*Cg*1^iii^	0.97	2.73	3.6159 (16)	152
C28—H28*A*···*Cg*2^iv^	0.97	2.93	3.8481 (16)	159

Symmetry codes: (i) −*x*+3, −*y*, −*z*+1; (ii) *x*−4, *y*+1, *z*−1; (iii) *x*−1, *y*, *z*; (iv) *x*−3, *y*+1, *z*−1.
